# Safety and Precision AI for a Modern Digital Health System

**DOI:** 10.1055/s-0044-1800714

**Published:** 2025-04-08

**Authors:** Elizabeth M. Borycki, Linda W. P. Peute, Femke van Sinderen, David Kaufman, Andre W. Kushniruk

**Affiliations:** 1 School of Health Information Science, University of Victoria, Victoria, British Columbia, Canada; 2 Department of Medical Informatics, Amsterdam UMC Location, University of Amsterdam Amsterdam, The Netherlands; 3 School of Health Professions Faculty, Health/Medical Informatics, SUNY Downstate Health Sciences University, Brooklyn, NY

## Abstract

Artificial intelligence (AI) promises to revolutionize healthcare. Currently there is a proliferation of new AI applications that are being developed and beginning to be deployed across many areas in healthcare to streamline and make healthcare processes more efficient. In addition, AI has the potential to support personalized and customized precision healthcare by providing intelligent interaction with end users. However, to achieve the goal of precision AI issues and concerns related to the safety of AI, as with any new technology, must be addressed. In this article we first describe the link between AI and safety and then describe the relation of AI to the emerging study of technology-induced error. An overview of published safety issues that have been associated with introduction of AI are described and categorized. These include potential for error to arise from varied sources, including the data used to drive AI applications, and the design process of AI applications itself. In addition, lack of appropriate and rigorous testing and limited analysis of AI applications during procurement processes has also been reported. Recommendations for ensuring the safe adoption of AI technology in healthcare are discussed, focusing on the need for more rigorous testing and evaluation of AI applications, ranging from laboratory testing through to naturalistic evaluation. The application of such approaches will support safety and precision AI for a modern digital health system.

## 1. Introduction

Healthcare is currently experiencing an Artificial Intelligence (AI) revolution. Recent developments and advances in AI are moving forward a new generation of health-focused software, hardware products and technologies that are making significant changes in the way that citizens and health professionals provide patient care [ 1 2 3 ]. AI technologies can improve wellness, screen for, and diagnose disease, as well as provide individually tailored health and disease specific interventions [ 1 2 3 ]. The future benefits of AI across health settings have the potential to improve patient outcomes while at the same time enhancing the quality and safety of healthcare. Biomedical and health informatics professionals, who are responsible for designing, testing, implementing, and managing AI technologies, need to consider the challenges and benefits, of using these new tools.

AI provides unprecedented opportunities to advance precision healthcare. AI can be used to tailor and customize healthcare advice and support. Such systems can provide patients with opportunities to receive customized care in a range of health settings and contexts [ 1 2 3 ]. However, health focused precision AI will need to be thoroughly designed, tested and managed to prevent inadvertent introduction of safety issues, when using the technology [ 1 , 4 ]. AI's precision is influenced by many factors including data quality, design, testing, procurement, implementation, privacy, security and its management [ 3 4 5 6 ].

The accuracy, correctness and appropriateness of such personalization using AI technologies will need to be carefully assessed and evaluated to ensure system safety is maintained from its initial use through to its obsolesce [ 3 , 4 ]. As AI has the potential to introduce new types of errors, there is a need to consider varying aspects of the technology to ensure its safety, when using AI in health settings. Such considerations include evaluating the algorithm for racial and ethnic bias [ 3 , 6 ] or fit with the context that it will be used in. Without such attention to the technology, users or healthcare organizations experience or provide ineffective, and/or inappropriate care ( *i.e.* , preventing the patient from receiving tailored, precision healthcare) [ 7 ]. Therefore, AI technology precision and its subsequent effects on safety is an important aspect of its real-world use in health settings. In this paper we review some of the key considerations that health and biomedical informatics professionals need to account for to allow for the safe design, integration, alignment and use of AI to support precision healthcare for patients.

More specifically, the objectives of this paper are to:

- Define AI;
- Define AI safety;
- Outline the link between precision alignment, health, healthcare processes and AI;
- Describe some of the health technology safety issues that we are currently experiencing in health care;
- Provide an overview of some of the published safety issues associated with the use of AI that have been introduced to health care;
- Suggest future research directions in this area of emerging research.

## 2. Background

AI's potential in healthcare has been described as revolutionary by many technology innovators. AI innovators identify the benefits and challenges that AI tools will provide to the healthcare system as significant; for example, Elon Musk has suggested that AI-Enabled Healthcare Innovation can improve “ *patient outcomes by providing more accurate diagnoses and treatment plans* ”. Healthcare providers can ensure that patients receive the best possible care. AI-enabled solutions can help healthcare providers identify high-risk patients and intervene before serious health issues arise [ 8 ]. Sam Altman, OpenAI's Chief Executive Officer, believes there is an expanded role for AI in providing healthcare. Altman, has proposed that AI will improve access to healthcare by providing medical advice to individuals, who are currently unable to pay for traditional health professional visits [ 9 ]. Both innovators believe that AI tools, when applied to healthcare, hold considerable promise as well as present important challenges, for the healthcare industry to address. The potential of AI advancements to provide precision healthcare may be significant and therefore biomedical and health informatics professionals need to develop a comprehensive approach to addressing safety issues so that individual and public health outcomes improve [ 2 , 3 , 8 9 10 ].

Yet, even as the potential of AI is being evaluated in varying healthcare settings, several serious concerns are being identified by academics and industry [ 2 , 3 , 8 9 10 ]. A thoughtful and cautious approach is being advocated by many academic leaders towards the use of such tools [ 4 , 11 ]. Leaders in the field of biomedical and health informatics are among those, who are identifying potential issues associated with AI's application and use in healthcare. These leaders are advocating for a greater understanding and evaluation of AI in the areas of safe design, development, implementation, and management [ 2 , 3 , 5 ]. In the next section of this traditional or narrative literature review [ 12 ] the authors consider the implications of AI upon safety. We begin by defining the concept and developing an understanding of some of the issues that fall within this important technology safety area.

## 3. Definition of AI

To best understand AI, it is important to first define what we mean by this technology. AI refers to “the theory and development of computer systems able to perform tasks that normally require human intelligence, such as visual perception, speech recognition, decision-making, and translation between languages.” (Oxford Languages and Google, nd. https://languages.oup.com/google-dictionary-en/ ). AI and its use have been and continue to be fundamental to how we currently provide healthcare. It must be noted that some AI tools have supported some aspects of health professional decision-making effectively for many years now [ 1 ].

AI has already improved patient safety. AI is being used in some healthcare settings and contexts with great success [ 1 ]. AI tools are currently being used to: (1) improve drug safety ( *i.e.* , prevent drug adverse events); (2) improve clinical reporting, and (3) enhance alerting and/or alarms that let health professionals know the patients' physiologic status is deteriorating. These AI software and hardware tools have improved the health professionals' abilities to provide high quality, accurate, and safe healthcare as well as patient outcomes [ 1 ]. Designers and developers of AI tools have worked to refine the “measurement, calculation, or specification” of care”. Such attention to improving AI with a focus on precision has been critical to deploying and improving healthcare using AI.

## 4. AI and Safety

Drug safety and adverse drug event alerting remain an important area, where AI tools have been implemented, for the purpose of improving patient safety. AI has been used to enable drug-drug interaction checking to prevent patients from receiving medications that may lead to human harm. AI tools have been used to monitor for and prevent patient safety events such as receiving the incorrect administration of the wrong medication to the wrong patient, providing the wrong dose of a medication to a patient (leading to either an overdose or an underdose of a medication), as well as ensuring that patients are prescribed their appropriate medications as they transition between health settings. To illustrate, AI has improved transitions from hospital to home and hospital to long term care [ 1 ]. AI tools are being used to improve clinical reporting and health technology professionals' responses to safety events. AI has been used to identify safety incidents and to provide enhanced feedback to patients about their healthcare [ 1 , 13 , 14 ].

Laboratory test results and vital sign data have been analyzed using AI tools and technologies. AI has improved health professionals' review and use of laboratory test results and patient vital sign data in health focused decision making. This use of AI supports clinician diagnosis of disease and the selection of the most appropriate patient treatment approach. AI research has also included extraction and analyses of data found in electronic health records. AI technologies have demonstrated their value in identifying individuals, who are at risk for bleeding, surgical complications (post-operatively), mortality, and other health events before they occur [ 1 ].

AI has improved alarms and alerts. When triggered, these alarms and alerting mechanisms help health professionals to identify, when a patient's condition deteriorating so that a health intervention can be made. Alarms and alerts integrated with AI, signal to the health professional that the patient is about to experience a health event. Al has been used to improve alert and alarm performance (by enhancing the precision of health monitoring), and reduce the number and frequency of false alarms/alerts (especially in cardiac care, intensive care units and emergency departments). AI clinical alarm algorithms can help support health professionals' understanding of patient vital sign data, and the prediction of patient deterioration in health status and the development of adverse events [ 1 , 15 ].

In summary, many AI tools and technologies are used in healthcare settings to enhance patient outcomes and patient safety. AI tools have improved the diagnosis of disease, prescribing of medications and treatments, alerting of health professionals, and monitoring of vulnerable patients, who may need additional medical attention. AI has improved the precision of healthcare. Even so, AI related safety issues remain a concern for many AI innovators, health professional users and designers. There persists a need to continually improve AI technologies using a learning health systems approach (i.e., where data and health experience used in conjunction with technology is systematically used to inform and integrate new innovations and knowledge in healthcare settings) [ 16 ]. There is a continual need to improve the quality and safety of the AI tools (as with other technologies used in healthcare) to ensure a precise fit between the healthcare systems, where the AI is deployed, and the people and processes that use them [ 3 , 6 , 16 ].

## 5. Health Technology Safety Issues

Technology safety has emerged as an important consideration for citizens and health professionals, who use and work in a modern, digital healthcare system [ 17 18 19 ]. Researchers from around the world have identified that technology plays a significant role in: (1) improving the safety of healthcare; and (2) introducing new types of safety concerns (i.e., technology-induced errors) to the healthcare system. In 2000 the Institute of Medicine published the report “To Err is Human”. At the time, research suggested that 98,000 Americans died because of medical errors. Healthcare specific technologies such as electronic health records and computerized order entry systems (i.e., the predecessors of modern-day ePrescribing systems) were found to improve patient safety by standardizing, streamlining, re-engineering, and supporting new technology-driven processes [ 20 ]. Suddenly, data could be extracted from these systems and analyses could be conducted that allowed one to identify health care quality and safety issues. These health technologies improved how health care was provided by organizations. Patients, healthcare professionals and health focused organizations benefited from technology driven innovations and improvements.

In 2005, we saw the gradual recognition of a new type of error (i.e., the technology-induced error). “Technology-induced errors are medical errors that arise from the design and development of a technology, its implementation and customization, interactions between the operation of a technology and the new work processes that arise from a technology's use, its maintenance, and the interfacing of two or more technologies used to provide or support health care activities” [ 19 , 21 ]. Conventional software testing methods (i.e., white box, black box and grey box testing) [ 22 ] did not identify technology-induced errors, as these types of error are often only detected, when the technology is subjected to real-world healthcare conditions and contexts. The urgency and complexity of real-world patient health situations “induce” the user to make an error [ 17 18 19 , 21 ].

Researchers found that with the digitization of healthcare that technologies could improve safety, while at the same introduce new types of safety issues such as technology-induced errors [ 17 , 19 , 20 , 23 , 24 ]. The phenomena were found to be pervasive across software, devices, and platforms (e.g., mobile phones that use mHealth applications, desktop/laptop computers that use electronic health records, and tablets devices that provide access to patient portals) [ 19 , 23 24 25 26 ]. Safety events and issues were being identified and reported by health professionals [ 23 , 24 ] and patients [ 27 ]. This was especially the case in specific settings, where there was a significant reliance on technology by health professionals to diagnose and treat life critical health events such as in intensive care units and/or emergency departments. There is greater vulnerability to technology-induced errors in such healthcare settings because of the complexity and criticality of patient health issues and conditions encountered by health professionals in these health contexts. Therefore, the number, complexity and integrated use of technologies to provide life saving care is higher, leading to more awareness and reports of such errors among health professionals [ 23 , 24 ].

There have emerged relationships between technology-induced errors and usability (i.e., software, hardware and devices) [ 19 ], workflows associated with their use [ 28 ] and mismatches between technology and organizational fit [ 29 ]. Technology-induced errors were also found to propagate across software, devices and digital ecosystems of care (e.g., from physician office to hospital), when systems are integrated to support exchange of information between software and devices [ 30 ]. Yet, many technology-induced errors are common across vendors and systems of care [ 31 ], and this has afforded researchers around the world the ability to study and classify many of these types of errors so that we can better understand them and develop methods to prevent their future occurrence [ 23 , 24 , 30 , 32 ]. By 2011 the Institute of Medicine recognized that “to achieve better health care, a robust structure that supports learning and improving the safety of health information technology is essential. Proactive steps must be taken to ensure that health information technology is developed and implemented with safety as a primary focus”. This approach has revolutionized our thinking around integrating new technologies into healthcare.

## 6. AI Safety Issues

Health care innovations continue to be developed and deployed. New AI tools are among them. They are being used to augment the quality, safety and precision of digital health ecosystems in providing patient care, while streamlining healthcare processes. To illustrate in 2018, the National Health Service (NHS) in the United Kingdom deployed a chatbot that was intended to provide health advice as well as route patients to a physician to receive a virtual care physician visit. The chatbot had significant potential to improve and streamline healthcare processes. Yet, the chatbot introduced opportunities for error; for example, the chatbot misinterpreted patient dialogue, provided inaccurate diagnostic advice, missed patient symptoms, and increased, unnecessary use of emergency services (that may have led to delays in care for other patients who needed medical attention) [ 33 ].

With the introduction of any new AI technology, there is a need to monitor its use and engage in a process of continual improvement – a learning health systems approach needs to be taken [ 7 , 16 ]. Health systems learning allows for improving the precision of the technology to be refined and improved over time, thereby reducing opportunities for errors to be introduced to our digital health ecosystems [ 7 ]. There is a need for all participants (technology users, health informatics professionals) to take part in testing, evaluation, and reporting ( *i.e.* , via incident reporting systems) of safety events [ 24 ]. As outlined earlier, AI is currently being used in some settings in healthcare and AI is being introduced to other health settings. There is a need to recognize that AI is already part of our digital health ecosystem, and its roles in healthcare will continue to expand [ 1 , 3 ]. New safety issues will emerge as new AI technologies are introduced. Recognizing and identifying potential safety issues is the first step to creating safer digital health ecosystems of care.

In the current wave of AI technology tool development, several safety issues have emerged and have been brought to the fore (see Table 1 ). These include errors focused around: (1) AI and data quality; (2) AI design; (3) AI testing; (4) AI procurement; and (5) AI implementation. Each area of safety focus identifies a few examples of safety challenges, where attention to the precision of the technology would improve safety (see Table 1 ).

**Table 1.** AI Technology Safety Issues [ 4 , 6 , 38 , 39 ].

AI and Data Quality	Use of non-representative data in creatin the algorithm
	Biased data (e.g., Racial, Ethnic and Cultural Bias) was used
	Discrepancies between the training and test data
	The size of the training and test data sets were insufficient
	Noisy or meaningless data and/or statistical outliers were not handled
	Users were allowed to control the data
AI Design	Wrong, insufficient or poor designer specification of AI tasks or objectives
	Designer specifications that unexpectedly lead to harmful or unexpected side effects or results.
	Designers that did not realize alternative solutions or methods could produce the same or better results
	Designers ignoring environment context leading to harmful side effects
	Designer indifference to variables that: may change in real-world or naturalistic environments; may be harmful if changed once deployed (i.e., lead to technology-induced errors once deployed
	Designers letting users control the learning process
	Designers overgeneralizing rules or application of population statistics to individuals
	Designer solutions that: maximized rewards associated with using the solution for users or organizations. The AI is gamed to achieve specific user or organizational outcomes
	Lack of AI Explainability, transparency or trust leading to users not understanding what the AI is doing
Testing Issues	Inadequate naturalistic testing in real-life contexts
	Failure to test the system in a new environment
	Inadequate or failure to test for AI: Accuracy Privacy Real-world conditions Rare or extreme conditions Reliability Robustness Security Usability Usefulness Workflow integration(s)
	Designers failing to test the AI and compare its outcomes to existing or alternative solutions to determine if the AI solution is better
Procurement	Validity of the algorithms for the implementation site was not determined
	Mismatches occur between what the AI can be used for and the context of use
Implementation	Deploying an underperforming system
	Poor integration or fit into an existing digital health ecosystem leading to an error
	Unintended uses leading to errors during adoption
	Deploying an underperforming system Deploying a system that is unable to adapt to changes in the environment. Failure to explain to users: How the technology works (Explainable AI) Its benefits Its limitations What to do if an error is encountered Failure to account for unintended uses of the system by users Strategies for sensitizing users to and monitoring for automation complacency
	Privacy issues were not considered (e.g., no security mechanism implemented to prevent nefarious actors from altering the tool)
	Privacy and security issues were not considered. Security issues may include nefarious actors: maliciously modifying data leading to misclassification, gross miscalculations, and incorrect predictions/outputs or decisions poisoning data by modifying or manipulating a portion of the dataset that the AI is being trained on, validated, and tested on leading to misclassification, systemic malfunction, and/or poor performance of the AI
	Vulnerability of where the data is stored. This could lead to privacy/security breaches
	The whole healthcare system where the AI tool is implemented was not evaluated to prevent unforeseen outcomes at another part of the system
	A lack of transparency such that information about the owner/vendor/software/algorithms is not accessible and cannot be used by users to make informed decisions about the systems' use

AI and data quality remain an important consideration. Insufficient data, biased data, meaningless data, statistical outliers in the data and inconsistencies in the training data are top concerns that affect the precision, quality and safety of AI, when integrated into digital health ecosystems [ 3 , 34 ]. AI tool design has also been identified as a concern. It has been noted in the literature that insufficient, poor, or wrong specification of AI tools may have serious implications for safety. Designer imprecision or failure to account for environmental or contextual changes ( *i.e.* , the location where the AI tool is being deployed) may have safety impacts. Designer over generalization of rules, inappropriate application of statistics, and the introduction of gamification to the AI tools' design may also lead to safety issues [ 6 ]. The precision of AI tools can be improved using a more fulsome testing approach prior to technology deployment. Testing is a critical aspect of AI technology safety; for example, inadequate testing or failure to fully test an AI technology may introduce new safety issues [ 4 5 6 ].

An emerging area of safety focus in the literature is the role of procurement processes in identifying AI tools that have a tight fit with existing organizational work processes and activities [ 6 ]. Procurement researchers recommend fulsome exploration of the fit of technology tools to each healthcare context prior to committing to software and/or hardware contracts [ 7 , 35 , 36 ]. According to some researchers, inadequate testing as part of procurement processes may introduce new technology safety issues. A variety of approaches can be employed as part of the procurement process from conventional vendor demonstrations through to the use of clinical scenarios developed by healthcare organizations, heuristic evaluation through to usability testing and onsite implementation in the form or test deployments [ 29 , 35 , 37 ]. Test deployments offer insights into the fit of the technology with the local healthcare digital ecosystem [ 29 , 35 ]. To illustrate, during the procurement process, AI algorithms could be validated for their fit and effectiveness in a local environment ( *e.g.* , country, regional health authority or hospital). Procurements that fail to determine algorithm validity or fail to determine if there is a match between the technology and the environment may introduce unnecessary safety risks arising from mismatches between the local environment and the technology [ 6 ].

AI implementors may influence safe use of AI. Poor AI tool-digital ecosystem fit, and a lack of attention to users (so that they can develop precise, in-depth knowledge of the technology) in terms of what the technology does, its potential benefits and limitations ( *i.e.* , when human review and decision making it critical) can have impacts on how the technology is used from a quality and safety perspective [ 38 ]. Research has found that attention to AI Explainability or what the technology does ( *i.e.* , greater transparency) improves clinician and patient trust in the technology as well as technology acceptance, adoption and safe use during implementation [ 6 , 38 ]. Furthermore, outlining potential pitfalls of the technology such as automation bias may sensitize users to the limitations of AI and prevent users from becoming overly reliant on the technology in supporting their decision making (especially as this may lead user to make errors in decision making in some patient circumstances). Implementing organizations should carefully consider the nature and types of user training in this area. Alerting users to the limits of the technology and, also some of the issues associated with its use over time should be a consideration [ 39 ]. Privacy and security considerations are essential, when deploying AI tools.

Like other technologies, nefarious actors ( *e.g.* , hackers) may alter the precision and performance of AI technologies and this may lead to safety issues for both the patient and the health professional user [ 6 ].

To ensure AI tools are safe, several key areas of focus have emerged. Specifically, there will be a need to consider AI and data quality, AI design, testing, procurement, and implementation as each of these aspects of AI may influence safety. AI represents a complex group of technologies that are being integrated into our modern digital healthcare ecosystems. Along these lines, more stringent testing and evaluation methods are needed to ensure the safety of AI applications and tools for use in complex healthcare settings. Approaches borrowed from human factors engineering and modified and extended to address areas where AI technology could inadvertently cause error need to be applied. This includes applying techniques that range from laboratory-based methods to simulation and naturalistic testing approaches. This may involve usability inspection methods, usability testing as well as clinical simulations to ensure both the usability and safety of AI. In addition, the evaluation of systems in near-live or naturalistic contexts is needed [ 14 ].

Over the past 20 years, these methodologies have been effectively applied as a means of identifying and addressing technology induced errors arising from use of a range of health information technologies, from electronic health record systems to advanced decision support tools [ 40 ]. A layered approach to applying these methods, where they are applied in sequence, starting with the evaluation of use of the technology under controlled artificial conditions, followed by error correction and the application of clinical simulation methods under near live or realistic naturalistic conditions is recommended. This may in turn be followed by testing of AI systems and applications under real-world naturalistic conditions prior to widespread deployment [ 14 ]. As we with any other complex health care technology, the adequate testing of new technologies under a range of conditions and using multiple methods will be essential to ensure the safety of AI applications. These methods have proven to be an effective means of identifying specific types of digital health ecosystem safety issues. Although some of the methods are specific to some types of technologies, others can be effectively used and modified specifically to understand the effects of introducing AI into differing types of care settings upon the quality and safety of the patient care encounter [ 7 ].

## 7. Conclusion

In summary, healthcare has moved in the last 30 years from a largely paper-based system of care through to a hybrid paper-electronic healthcare system to a highly digitized and increasingly interoperable and integrated system of digital ecosystem of care. Healthcare systems include electronic health records, patient portals, pharmacy information systems and electronic medical records whose reach extends to touch citizens who require healthcare in their homes, the workplace, the community and in hospital. With the introduction of new classes of applications based on AI we are now challenged with effectively integrating this technology in the healthcare system so that safety is a paramount consideration. To address this challenge, our understanding of the complex type of errors that may inadvertently be introduced by AI technology needs to be more fully considered. This may be considered in the context of where and how AI safety issues may initially arise ( *e.g.* , from procurement and design through to implementation). In addition, the standard application of a wider range of testing methods is needed before widespread deployment to ensure AI, as with any technology, does what it was designed, procured and implemented to do, and does not inadvertently induce error or compromise safety in healthcare. AI has the potential to advance and customize its advice and support of both healthcare providers and patients. This trend will make consideration of human factors and safety issues increasingly important as we move towards personalized and tailored AI advice and support through precision healthcare. With proper safeguards in place, AI can be a profoundly transformative technology in providing precision healthcare.
